# An Address

**Published:** 1863-07

**Authors:** S. B. Palmer

**Affiliations:** Tully, N. Y.


### AN ADDRESS.

BY DR. S. B. PALMER, OF TULLY, N. Y.

(Read at the first regular meeting of the Central New York Dental Association, held in Auburn, May 12th, 1863.)

*Mr. President and Gentlemen* :—As a result of perseverance, much labor and a number of preliminary meetings, we are to-day assembled in the capacity of a Dental Association in Central New York. And yet, all that has been accomplished thus far is but an experiment. Will this experiment succeed, and we, as dentists, derive the benefit of associated labor, and social intercourse, or fail, and blast the bright hopes now before us, and thereby assign to each his accustomed routine of labor, and apportionate, to still go on plodding round and round in the same selfish circle, is a matter of time, which the future alone can reveal.

In either case the effort will not be unlike others that have preceded us. On one hand we see but the record left of a like organization, on the other a living promising association.

Perhaps the only distinction to be made between this and former meetings of this society consists in appointing the present the first regular meeting of this Association. When years shall have passed we may revert to this day and and its transactions with disappointment and regret, or with satisfaction and pleasure, as, on similar occasions, we meet to refresh and improve our minds professionally or to gladden and cheer our hearts socially.

The ultimate success of this enterprise depends wholly upon individual effort to sustain and make each meeting interesting and instructive.

Let us, as far as possible, carry out the sentiment of that article in the constitution which says "The object of this Association shall be for mutual improvement and professional intercourse of its members."

Dentistry arose from a position akin to the occupation of a common barber, and is now one of the learned professions of the present day.

How has this rapid change been accomplished? Not by selfishly hoarding up for individual promotion all new and useful discoveries.

The early history of the science marks the improvement under such influence, and its growth appears much like some kinds of timber found growing in tropical climate, whose annular rings can hardly be traced.

By the establishing of Dental Colleges, Dental Periodicals and Dental Associations, we behold progress in the science of dentistry in America unequalled by any other nation or people.

The present, how unlike the past, when the dental student could be regarded as one "in pursuit of knowledge under difficulties!"

The "No Admittance" card greeting him at the door of nearly every laboratory, and for the detached portion of the science and art then in market, compelled to pay a price as extravagant as the knowledge was secret.

But, thanks to the pioneer and the liberal minded of the profession, they have left for us a rich legacy of discovery, experiment and experience.

True, many of the earlier modes of manipulating have been superceded by modern discoveries; and the liberal exchange of ideas conveyed through the various periodicals devoted to the science have served to render the means of acquiring a thorough knowledge of dentistry comparatively easy. Do all who claim the title of dentist possess such knowledge?

With all the facilities afforded for instruction, we see a vast difference in the various operations performed by different members of the profession. Why this difference? and why so many inferior operations? Perhaps a book of knowledge and skill to operate correctly, perhaps for want of proper designs or patterns to imitate, or intimation that others could do even better.

In a science like dentistry which embraces various divisions of both mechanics and the arts the mind has opportunity, and very naturally seeks and finds its affinity in some one division more than another, and as minds vary as well as subjects, no department is likely to escape special attention.

One of the advantages of the association is, to bring together the result of those special labors and experiences in the several apartments, and to compile and make one whole record of the knowledge thus obtained, for the benefit of each and all.

Mankind are but creatures of imitation, and the so-called "genius but an example of unyielding perseverance." How important, then, that we have the best lessons and designs set before us for imitation.

Although at present we may not be able to command that degree of instructive talent of which older societies can boast, yet we can make this a nucleus around which to rally a recruiting station, where we may enroll our names as volunteers for professional improvement and public service.

The subject treated at our last meeting was convincing to all that dentists have *pecuniary* claims upon their patrons; let us in charity acknowledge that our patrons also have equivalent claims upon us.

Mere titles amount to nothing; and the advertisements of the empiric so far exceed those of the proficient, unpretending practitioner, that even a meritorious discovery or improvement announced by the latter, if in advance of the usual adopted modes, is looked upon with scepticism and distrust.

The sign of "Dentist," placed at the entrance of an office, should be a sufficient guaranty to the unfortunate in search of the professional service, that he might expect the service of one skilled and experienced in the dental art.

Does that title, or one more highly qualified, give any such assurance? Could we see a graduated scale on which was marked the various degrees of dental talent, it would be found to exceed in length the scale of a Farenheit thermometer.

In the science of medicine all are doctors from the most eminent and skillful, down to those whose highest attainments could only admit of prescribing catnip tea to a restless infant, recommend carrying in the pocket a small swine-bone as an infallible cure for rhuematism.

So in our profession all are dentists, from those possessing the highest degree of merit, down to those whose greatest success mainly consists in the free use of *plastic materials*, whose only preficiency in art is manifested in extracting unjust fees from their patrons pockets.

Gentlemen, we don't expect to correct all these evils; but let us unite in drawing a line of distinction between the worthy and unworthy—the skillful and the impiric.

Are not dentists themselves responsible for introducing, or inducing into the profession, many pretended practitioners, who are, and ever will be, mere clogs to the wheels of progress? Have we not measured the blocks which we have fitted to take place in the great dental monument by a rule too short? Are we sure that they may be placed in the structure without the sound of a hammer?

Let us compare our rules with the universal standard, and, when in harmony with them, then will they be in harmony with each other.

Let us say to the aspirant in search of a lucrative employment, who is desirous of learning the "Dental Trade" in one year or less, "Dear Sir: Dentistry 'has viz:' we cannot now, as in years past, prepare students and send them out as proficient dentists even in *three* years. There are colleges instituted for the purpose of instructing dental students in the science and art of the profession which afford opportunities not usually found in private practice."

Does such an answer discourage the inquirer? The dentist giving such information has but performed his duty to the applicant, to the profession, and perhaps saved an untold amount of mischief to the public.

Another two-fold evil exists for which dentists and their patients are alike responsible, viz: The reduction of dentists' fees below a standard that will stimulate the operator to put forth his best efforts for success. It is a well known fact that in any branch of industry, the most competent and skillful workman can command the highest comparative wages. We say comparative on account of the impossibility of establishing a universal price list to suit all localities and conditions of people.

The dentist who may receive five dollars for professional service in one of our large cities would find more difficulty to obtain two dollars in this city, or even one dollar in a remote rural district. Notwithstanding this, no dentist is doing honor to himself or justice to his patient who consents to labor for a pitance so small as to deprive himself of the opportunities of experiment, reading or other means necessary for professional improvement.

Because our patients demand of us cheap dentistry, and go dental shopping to find a dealer in the article, that is no reason we should accept their proposals and consent to render service for a remuneration so small that, to perform the task well, would be a sacrifice of time on our part, or, in the attempt to make a profit by like operation, be compelled to manipulate in an unwarrantable degree of slight and haste.

Gentlemen, as members of this Association, let us present a bold front to these growing evils, learn what is our duty, and "stand for the right."

If we cannot adopt one standard of prices, let us know the minimum. There is a zero in the scale of each member, below which to settle would be disrespectful to the operator and to the profession, and, in most cases, detrimental to the patient. This matter of price rests with the profession. As the price of merchandise conforms to the premium on gold, so will the efficiency of dental labor conform to the facilities afforded for experiment, investigation and improvement.

The dentist, possessing proper respect for his own reputation or the good of his patrons, can but feel that he "must be a man of science as well as a mechanic;" and, unless he is willing to avail himself of every means within his reach to obtain such knowlege, is not worthy of the confidence of the profession or the patronage of the public.

And in conclusion, as we have formed an Association, and given our names as members of the same, let us cast aside all professional jealousy that may have existed from the imperfect knowledge we have of each other, as gathered from price biding or disaffected patient, and hereafter cherish towards each others feelings of friendship and respect.

Let us be slow to believe all the delaterious reports we may hear against the honor and integrity of our brother in the profession, remembering that derogatory reports, like rolling snow balls, lose nothing by the journey. And while this Association may become to its members "a dental school, its members acting as teacher and pupils," let it also be a school of professional etiquette, setting forth examples of friendship, forbearance and forgiveness.

